# Effect of Time-Restricted Eating on Circulating Levels of IGF1 and Its Binding Proteins in Obesity: An Exploratory Analysis of a Randomized Controlled Trial

**DOI:** 10.3390/nu16203476

**Published:** 2024-10-14

**Authors:** Rand Talal Akasheh, Aparna Ankireddy, Kelsey Gabel, Mark Ezpeleta, Shuhao Lin, Chandra Mohan Tamatam, Sekhar P. Reddy, Bonnie Spring, Ting-Yuan David Cheng, Luigi Fontana, Seema Ahsan Khan, Krista A. Varady, Sofia Cienfuegos, Faiza Kalam

**Affiliations:** 1 Department of Nutrition and Dietetics, American University of Madaba, Madaba P.O. Box 2882, Jordan; 2 Division of Cancer Prevention and Control, Department of Internal Medicine, The Ohio State University, Columbus, OH 43214, USAfaiza.kalam@osumc.edu (F.K.); 3 Department of Pediatrics, University of Illinois Chicago, Chicago, IL 60607, USAsreddy03@uic.edu (S.P.R.); 4 Department of Kinesiology and Nutrition, University of Illinois at Chicago, Chicago, IL 60607, USAslin89@uic.edu (S.L.);; 5 Department of Preventive Medicine, Northwestern University Feinberg School of Medicine, Chicago, IL 60611, USA; 6 Charles Perkins Centre RPA Clinic, Sydney Medical School, University of Sydney, Sydney 2009, Australia; 7 Department of Endocrinology, Royal Prince Alfred Hospital, Camperdown 2050, Australia

**Keywords:** intermittent fasting, obesity, growth hormones, IGF1, IGFBP, insulin

## Abstract

Obesity is associated with alterations in circulating IGF1, IGF1-binding proteins (IGFBPs), insulin, inflammatory markers, and hormones implicated in cardiovascular disease, diabetes, cancer, and aging. However, the effects of 4 and 6 h time-restricted eating (TRE) on circulating IGF1 and IGFBPs is uncertain. Objective: This study aimed to investigate the effects of TRE on plasma IGF1, IGFBP1, IGFBP2, and IGFBP3, and whether these effects were mediated by weight loss or body composition changes. Insulin sensitivity, glucose control, adipokines, and inflammatory markers were also examined. Design: An exploratory analysis of an 8-week randomized controlled trial implementing a daily TRE intervention was carried out. Participants/Setting: This study was conducted at the University of Illinois at Chicago in 2019. Participants with obesity were randomized to 4 or 6 h TRE (n = 35) or a control (n = 14) group. Plasma biomarkers were measured by ELISA at baseline and week 8. In a sub-analysis, participants were stratified into higher- (>3.5%) and lower- (≤3.5%) weight-loss groups. Intervention: Participants fasted daily from 7 p.m. to 3 p.m. in the 4 h TRE group (20 h) and from 7 p.m. to 1 p.m. in the 6 h TRE group (18 h), followed by ad libitum eating for the remainder of the day. Controls received no dietary recommendations. Main outcome measures: IGF1, IGFBPs, hsCRP, and adipokines were the main outcome measures of this analysis. Statistical Analysis: Repeated measures ANOVA and mediation analysis were conducted. Results: Body weight significantly decreased with TRE (−3.6 ± 0.3%), contrasting with controls (+0.2 ± 0.5%, *p* < 0.001). Significant effects of TRE over time were observed on plasma IGFBP2, insulin, HOMA-IR, and 8-isoprostane levels, without affecting other biomarkers. In the sub-analysis, IGFBP2 increased while leptin and 8-isoprostane decreased significantly only in the “higher weight loss” subgroup. Changes in insulin and HOMA-IR were related to TRE adherence. Conclusions: Eight-week daily 4 to 6 h TRE did not affect IGF1, IGFBP1, or IGFBP3 levels but improved insulin, HOMA-IR, and 8-isoprostane. IGFBP2 increased and leptin decreased when weight loss exceeded 3.5% of baseline.

## 1. Introduction

Time-restricted eating (TRE) is an emerging form of intermittent fasting associated with caloric restriction conducive to weight loss, with potential benefits for cardiovascular disease, diabetes, and cancer prevention. It involves consuming meals within a specific time window and fasting for the remainder of the day [1]. Obesity increases the risk of several common malignancies, including breast, colon, endometrial, and liver cancer, among others. The pathogenesis involves obesity-induced insulin resistance, compensatory hyperinsulinemia, and changes in sex hormones and insulin-like growth factor-1 (IGF-1) bioavailability, alongside elevated inflammation and oxidative stress. Consequently, interventions targeting these biomarkers are expected to mitigate cancer risk [2]. 

Indeed, the well-documented cancer-preventive effects of caloric restriction are partially attributed to inhibiting the protein kinase B (AKT)/mammalian target of rapamycin (mTOR) pathway through reduced circulating levels of insulin and IGF1, along with increased levels of IGF1-binding protein 1 and 2 (IGFBP-1 and 2) [3]. Importantly, IGFBPs refer to a family of proteins that bind IGF1 and prevent its binding to its receptors, thereby limiting IGF1 bioavailability and physiological activity. Calorie restriction can also reduce cancer risk by inducing a powerful reduction in systemic inflammation, adipokine changes such as decreased leptin and increased adiponectin, and a decrease in sex hormone levels and bioavailability [3].

Evidence suggests beneficial effects of TRE on glucose, insulin, oxidative stress, leptin, and adiponectin levels, indicating cardioprotective properties [4,5,6,7,8,9]. Additionally, the extended fasting time in TRE regimens is expected to reduce the exposure to cancer-promoting growth factors like insulin and IGF-1. We previously demonstrated the effect of 4 to 6 h TRE intervention on cardiometabolic indicators [4], sex hormones, and sex hormone-binding globulin [10]. However, reports on how TRE influences circulating IGF1 and its binding proteins are limited and inconclusive. For example, serum IGF1 levels increased in elite athletes [11,12] but decreased in women with PCOS following 8 h TRE interventions [13]. However, to our knowledge, the effects of 4 to 6 h TRE on IGF1 have not been investigated. Additionally, weight loss and calorie restriction can increase circulating levels of IGFBPs [14,15]. For example, a two-year intervention entailing 25% caloric restriction for participants with normal weight increased serum IGFBP1 by 21% and decreased the IGF1/IGFBP1 ratio by 42%. Another six-month clinical trial for women with overweight found that 25% continuous as well as intermittent caloric restriction exerted comparable weight loss and increased IGFBP1 and IGFBP2 levels. However, the effects of TRE on circulating levels of IGFBPs are yet to be elucidated.

To address these research gaps, this study investigated the impact of daily 4 to 6 h TRE on the circulating levels of growth factors, IGFBPs, adipokines, and inflammatory markers. We hypothesized that daily 4 to 6 h TRE exerts favorable changes in circulating IGF1, IGFBP1, IGFBP2, and IGFBP3, in addition to insulin, leptin, adiponectin, 8-isoprostane, and high-sensitivity C-reactive protein (hsCRP). Moreover, this study aimed to determine whether the potential benefits of TRE on these biomarkers were, wholly or partly, mediated by caloric restriction and the resulting weight loss. A sub-analysis was conducted to investigate whether the effects of TRE on circulating growth factors, glucoregulatory indicators, adipokines, and inflammatory markers were dependent on weight loss. We hypothesized that some of the changes exerted by TRE on these biomarkers are independent of weight loss.

## 2. Methods

### 2.1. Recruitment of Study Participants

The Office of the Protection of Research Subjects at the University of Illinois at Chicago (UIC) approved the protocol. The full experimental procedures have previously been published [4]. This current study constitutes an exploratory post-hoc analysis of a 10-week randomized parallel-arm trial investigating the impact of 4 to 6 h TRE vs. control on body weight and metabolic health in adults with obesity [4]. The study was conducted in 2019 in the laboratories of the Department of Kinesiology and Nutrition at UIC. Participants were recruited from the Chicago area through advertisements announced throughout UIC campus. A questionnaire, body mass index (BMI) assessment, and pregnancy test were used to screen participants for eligibility. All participants provided written informed consent before being enrolled in the study. The inclusion criteria comprised being male or female, between the ages of 18 and 65, with a BMI of 30 to 49.9 kg/m^2^. Conversely, individuals who smoked, had a history of diabetes, were pregnant, lactating, or were not weight-stable were excluded. Details regarding sample size calculation, randomization, blinding, and intervention safety were reported in the published protocol [4]. Demographic information including age, sex, and race/ethnicity were self-reported by participants. The primary analysis of this trial revealed no differences between the effects of 4 vs. 6 h TRE on metabolic parameters [4]. Thus, completers in the 4 and 6 h TRE groups (n = 16 and 19, respectively) were combined into one TRE group (n = 35) and compared with controls (n = 14) [4]. 

### 2.2. Time-Restricted Eating Protocol

Participants underwent a two-week weight-stabilization period at the baseline, followed by an eight-week intervention period. Participants on TRE were allowed to eat freely during the eating window but were restricted to water and energy-free beverages during fasting. Participants in the 4-h TRE group fasted daily for a total of 20 h from 7 p.m. to 3 p.m. the next day, while in the 6-h TRE group they fasted for a total of 18 h from 7 p.m. to 1 p.m. the next day. Participants were instructed to maintain their usual levels of physical activity throughout the trial. The study flow chart is available in the original publication [4]. 

### 2.3. Control Group Protocol

Controls received no dietary recommendations and were advised to maintain their usual eating and exercise routines. To minimize potential bias, controls visited the research facility as frequently as the intervention groups. Completing the 10-week trial entitled controls to four complimentary weight loss counseling sessions [4].

### 2.4. Body Weight, Body Composition, Diet Adherence, and Physical Activity

All outcomes for TRE and control groups were measured at baseline (pre-intervention) and week 8 (post-intervention). A HealthOMeter digital scale (Boca Raton, FL, USA) was used to measure body weight. Dual X-ray absorptiometry (DXA; iDXA, General Electric, Healthcare, Madison, WI) was used to assess fat mass, lean mass, and visceral fat mass. Each participant reported on a daily log the times they started or stopped eating, in order to count the number of TRE adherence days per week, as previously described [4]. Physical activity changes in terms of step count per day were evaluated over 7 days using a Fitbit Alta HR (Fitbit, San Francisco, CA, USA). 

### 2.5. Measurement of Circulating Factors

Twelve-hour fasting blood samples were collected between 6 a.m. and 9 a.m. at baseline and following the eight-week intervention. Plasma was extracted as previously described [4]. The participants were instructed to avoid exercise, alcohol, and coffee for 24 h before each visit. Concentrations of total IGF1, IGFBP1, IGFBP2, IGFBP3, leptin, high molecular weight (HMW) adiponectin, hsCRP, interleukin-6 (IL6), and tumor necrosis factor (TNF) were measured in duplicate by ELISA (Biotechne, Minneapolis, MN, USA). An ELISA kit purchased from Cayman Chemical (Ann Arbor, MI, USA) was used to measure 8-isoprostane. Concentrations of IGF1 and IGFBP3 in ng/mL were converted into nmol/L values to calculate the molar ratio of IGF1/IGFBP3 as a proxy for measuring free IGF1 levels. Fasting blood glucose, percentage of glycated hemoglobin A1C (HbA1C%), and plasma insulin concentrations were measured in a commercial lab (Medstar, Chicago, IL, USA). Homeostasis model assessment of insulin resistance (HOMA-IR) was calculated using the following equation:

[HOMA-IR = Fasting insulin (μIU/mL) × Fasting glucose (mg/dL)/405].

### 2.6. Sub-Analysis Comparing Participants with Lower-Weight Loss vs. Higher-Weight Loss

Post-intervention weight loss percentage relative to baseline was calculated for each participant. The TRE intervention resulted in an average weight loss of 3.5%. This was used as a cutoff to stratify all participants into the lower-weight-loss (≤3.5%) or higher-weight-loss (>3.5%) groups. Using this cutoff resulted in 29 participants in the lower- weight-loss group and 20 participants in the higher-weight-loss group. 

### 2.7. Statistical Analysis

Data were analyzed using JAMOVI software (version 2.3.21) [16]. Baseline differences between TRE vs. control participants were tested using an independent samples *t*-test (continuous variables) or McNemar test (categorical variables). Repeated measures ANOVA was conducted to compare changes in variables between groups (TRE vs, control, or higher vs. lower weight loss) over time (baseline and week 8). Pairwise multi-comparisons between groups and within the groups were performed to detect significant differences between any group–time combination. Additionally, repeated measures ANOVA was conducted before and after covariate adjustment, to explore whether the effect of group over time on biomarker levels was modified when adjusting for a covariate. The covariates used, each in a separate model, were TRE adherence, steps count, and the percentage of changes from baseline in body weight, waist circumference, and fat mass. Pearson’s product correlation coefficients were calculated in order to evaluate the associations between biomarker changes vs. weight and body composition changes over time. 

### 2.8. Mediation Analysis

To test whether weight loss or body composition changes mediated the effect of TRE on biomarker changes over time, generalized linear model (GLM) mediation analysis was conducted using the medmod package for Jamovi and R [16]. Data are presented as effect estimates ± standard error. The standard errors (SE) were calculated using the standard Delta method. Additionally, completely standardized effect sizes (β-coefficients) were calculated with Z-scores. *p*-values of <0.05 imply statistical significance.

## 3. Results

### 3.1. Baseline Characteristics

At baseline, no significant differences were observed between groups (TRE vs. control) for sex, race, age, height, body weight, BMI, waist circumference, fat mass, visceral fat mass, or lean mass (Table 1). However, TRE participants were significantly less physically active than controls, as measured in step count per day (7520 ± 507 vs. 9477 ± 795 steps/day, *p* < 0.05).

In the sub-analysis comparing lower- vs. higher-weight-loss participants, no baseline differences were observed for body weight, BMI, fat mass, visceral fat mass, lean mass, or weight circumference.

### 3.2. Anthropometric Changes after 8 Weeks of Time-Restricted Eating Intervention

Over the course of eight weeks, TRE caused a small but statistically significant reduction in body weight, BMI, waist circumference, fat mass, visceral fat mass, and lean mass (all *p*-values < 0.05). This aligned with high adherence to TRE, as reflected by a mean of 6.25 ± 0.10 days of fasting per week in the TRE group. Additionally, at baseline, TRE participants were significantly less active than controls, and this distinction persisted throughout the intervention, with no significant changes in step counts observed over time for either group (Table 1). 

**Figure 1:**
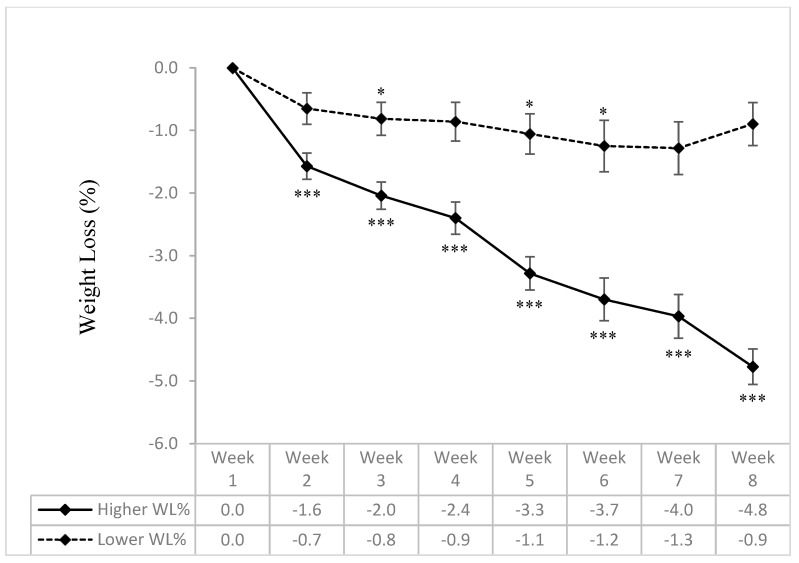
Percentage of weight loss relative to baseline, stratified according to final weight loss percentage (lower vs. higher) over 8 weeks of time-restricted eating intervention. Data are expressed as mean ± SEM for percentage of weight loss (WL%) relative to baseline body weight. Participants were stratified into lower WL% (≤3.5%, n = 29) and higher WL% (>3.5%, n = 20). * *p* < 0.05 and *** *p* < 0.001 for mean weight loss percentage in a specific week vs. group-matched baseline.

For the sub-analysis, participants were categorized into two groups based on their weight loss percentages: ≤3.5% (lower weight loss) and >3.5% (higher weight loss). As expected, a significant group × time interaction effect on the change in body weight following the eight-week intervention was observed (interaction *p*-value < 0.001). The change in body weight at week 8 compared with baseline was statistically significant in the higher-weight-loss group (*p*-value < 0.001) but not in the lower-weight-loss group (Figure 1). Furthermore, no significant differences between groups, within groups, or in terms of group × time interaction were observed in the step counts among participants with high vs. low weight loss.

### 3.3. Changes in Plasma IGF1 and IGF-Binding Proteins after 8 Weeks of Time-Restricted Eating Intervention

*TRE* vs. *Control:* No significant effects of group over time were observed on circulating IGF1, IGFBP1, and IGFBP3 among TRE or control participants. Likewise, no significant effects of time or group-over-time interaction were observed to affect the IGF1/IGFBP3 ratio. However, a significant effect of group on the IGF1/IGFBP3 ratio was noted, as controls exhibited a higher IGF1/IGFBP3 ratio compared with TRE participants. The multi-comparison test showed that controls exhibited a significantly higher IGF1/IGFBP3 ratio compared with the TRE group at week 8 (Table 2). Moreover, adjusting for covariates like TRE adherence, step count, or changes in weight, fat mass, or waist circumference did not modify these results. 

Interestingly, a significant effect of group over time emerged concerning plasma IGFBP2 levels following the eight-week intervention (interaction *p*-value = 0.022); IGFBP2 increased in TRE participants (+36.0 (22.7) ng/mL, contrasting with a decrease in controls (−61.3 (33.0) ng/mL). However, these changes were not statistically significant in the multi-comparison analysis (*p*-value > 0.05 for both groups compared with their baselines) (Table 2). After repeating the analysis incorporating covariate adjustments, the significant interaction effect on IGFBP2 levels disappeared when accounting for factors such as TRE adherence or percentage changes in anthropometrics including body weight, waist circumference, or fat mass (all interaction effects became non-significant, *p*-values > 0.05), but not when adjusting for step count.

*Lower* vs. *higher weight loss comparison:* In the sub-analysis, no significant effects of group over time were observed on circulating IGF1, IGFBP1, and IGFBP3 among participants with either lower or higher weight loss (all interaction *p*-values > 0.05). Likewise, no significant effects of time, group, or interaction were observed regarding the IGF1/IGFBP3 ratio. A significant effect of group over time on plasma IGFBP2 levels was observed (interaction *p*-value = 0.019), with IGFBP2 levels significantly increasing in the higher-weight-loss group (+67.1 (31.1) ng/mL, *p*-value < 0.05) but non-significantly decreasing in the lower-weight-loss group (−29.9 ± 23.2 ng/mL, *p*-value > 0.05) after the eight-week intervention (Table 3). 

Further analysis revealed that changes in IGFBP2 levels significantly and negatively correlated with changes in BMI, body weight, and fat mass (*p*-value < 0.05) (Supplementary Table S1). 

Taken together, these data indicate that eight weeks of 4 to 6 h TRE did not affect circulating levels of IGF1, IGFBP1, and IGFBP3, or the IGF1/IGFBP3 ratio. The increase in IGFBP2 after the eight-week TRE intervention was significant only in the higher-weight-loss group but not the lower-weight-loss group. Moreover, changes in circulating IGFBP2 concentrations were related to weight loss and changes in fat mass.

### 3.4. Changes in Glucoregulatory Indicators after 8 Weeks of Time-Restricted Eating Intervention

TRE vs. control: A significant effect of group over time on plasma fasting insulin levels was detected (interaction *p*-value < 0.001). Post-intervention, fasting insulin levels significantly decreased in TRE participants (−2.24 (0.98) μIU/mL, *p*-value < 0.05) but rose in controls (+4.75 (1.66) μIU/mL, *p*-value < 0.01) (Table 2). Remarkably, the interaction effect on insulin persisted even after adjusting for weight loss, changes in body composition parameters, or step count. Moreover, a significant effect of group (TRE vs. control) over time on HOMA-IR was observed (interaction *p*-value < 0.001). While HOMA-IR scores significantly increased in controls compared with their baselines (+1.363 (0.49), *p*-value < 0.05), they decreased in TRE participants following the intervention (−0.648 (0.290), *p*-value < 0.05), indicating that TRE improved insulin sensitivity. The average HOMA-IR of TRE participants was reduced to 2.79 (0.38) after the intervention, almost reaching the normal cutoff of insulin sensitivity (HOMA-IR < 2.5). Adjusting for weight loss, body composition changes, biomarker changes, or step count did not change the significant effect of TRE on HOMA-IR (Table 2). However, the significant effect of TRE on insulin and HOMA-IR disappeared after adjusting for TRE adherence (*p*-values > 0.05).

No significant effect of TRE over time was observed on fasting blood glucose or HbA1C (interaction *p*-values > 0.05, respectively). However, time was observed to have a significant effect on HbA1C levels (*p* < 0.001), whereby HbA1C levels significantly decreased in TRE participants (−0.23% (0.05%), *p*-value < 0.001) and in controls (−0.21% (0.08%), *p*-value < 0.01) after eight weeks. With these decreases, HbA1C levels became lower than the 5.7% threshold for prediabetes diagnosis in both TRE and control groups.

Lower vs. higher weight loss comparison: Despite the changes in insulin and HOMA-IR correlating with weight loss percentage (Supplementary Table S1), no significant effect of weight loss percentage (higher vs. lower) over time was detected on insulin levels, fasting blood glucose, and HOMA-IR (interaction *p*-values > 0.05) (Table 3). However, the multi-comparison test showed that fasting blood glucose levels significantly decreased in higher-weight-loss participants (−6.364 (3.06) mg/dL, *p*-value < 0.05). In addition, a significant effect of time on HbA1C levels was detected (*p*-value < 0.001), but no significant group or interaction effects were observed, as HbA1C levels significantly decreased after 8 weeks in both higher- and lower-weight-loss participants compared with their baselines (Table 3). 

These findings suggest that TRE significantly improved measures of insulin sensitivity, causing reductions in insulin levels and HOMA-IR. Moreover, these changes were not significant after adjusting for TRE adherence.

### 3.5. Changes in Plasma Adipokines after 8 Weeks of Time-Restricted Eating Intervention

TRE vs. control: After the eight-week intervention, no significant effects of group, time, or interaction were observed regarding plasma leptin and HMW adiponectin concentrations (Table 2). 

Lower vs. higher weight loss comparison: A significant effect of group over time was observed on leptin levels (interaction *p*-value = 0.044), whereby plasma leptin levels decreased as expected in the higher-weight-loss group compared with its baseline (−18.1 (8.7) ng/mL, *p*-value < 0.05) in the multi-comparison test, but non-significantly increased in the lower-weight-loss group (+4.5 (6.3) ng/mL, *p*-value > 0.05). This parallels the greater reduction in fat mass observed in higher-weight-loss compared with lower-weight-loss participants. In addition, no significant effects of group, time, or interactions were observed regarding plasma HMW adiponectin levels (Table 3).

Overall, these findings indicated no significant effects of TRE over time on plasma leptin and HMW adiponectin levels. However, leptin levels significantly decreased following the intervention compared with baseline only in the higher-weight-loss group and not the lower-weight-loss group. This aligns with the observation that the change in plasma leptin significantly correlated with percentage of weight loss (Supplementary Table S1).

### 3.6. Markers of Inflammation and Oxidative Stress (TRE vs. Control)

No statistically significant effects of group, time, or interactions were observed regarding plasma hsCRP levels. However, a significant effect of group over time emerged concerning plasma 8-isoprostane concentration (p-interaction = 0.007), as 8-isoprostane significantly decreased over time in TRE (−12.00 ± 2.74 pg/mL, *p*-value < 0.001) but not in control participants (+2.70 ± 4.34, *p*-value = 0.05) according to the multi-comparison test. The effect of TRE on 8-isoprostane did not remain significant when adjusting for changes in body weight, waist circumference, or TRE adherence. Still, its significance persisted when adjusting for other variables. This suggests that changes in plasma 8-isoprostane are likely to have been influenced by TRE adherence and anthropometric changes (Table 2). 

Lower vs. higher weight loss comparison: No significant effects of group (higher vs. lower weight loss), time, or interactions were observed on hsCRP levels. However, a significant effect of group over time was observed on 8-isoprostane levels (interaction *p*-value = 0.048). Post-hoc analysis revealed that 8-isoprostane levels significantly decreased in the higher-weight-loss participants following the intervention (−13.93 ± 3.86 pg/mL, *p*-value < 0.01) but not in the lower-weight-loss participants (−3.71 ± 3.15 pg/mL, *p*-value > 0.05) (Table 3).

These findings suggest that TRE caused a significant reduction in 8-isoprostane levels. In the sub-analysis, the change in 8-isoprostance was significant only with high weight loss (>3.5%), corresponding with significant correlations between changes in 8-isoprostane with changes in body weight, fat mass, and waist circumference (Supplementary Table S1).

### 3.7. Mediators of the Effects of TRE on Circulating Biomarkers

**Figure 2:**
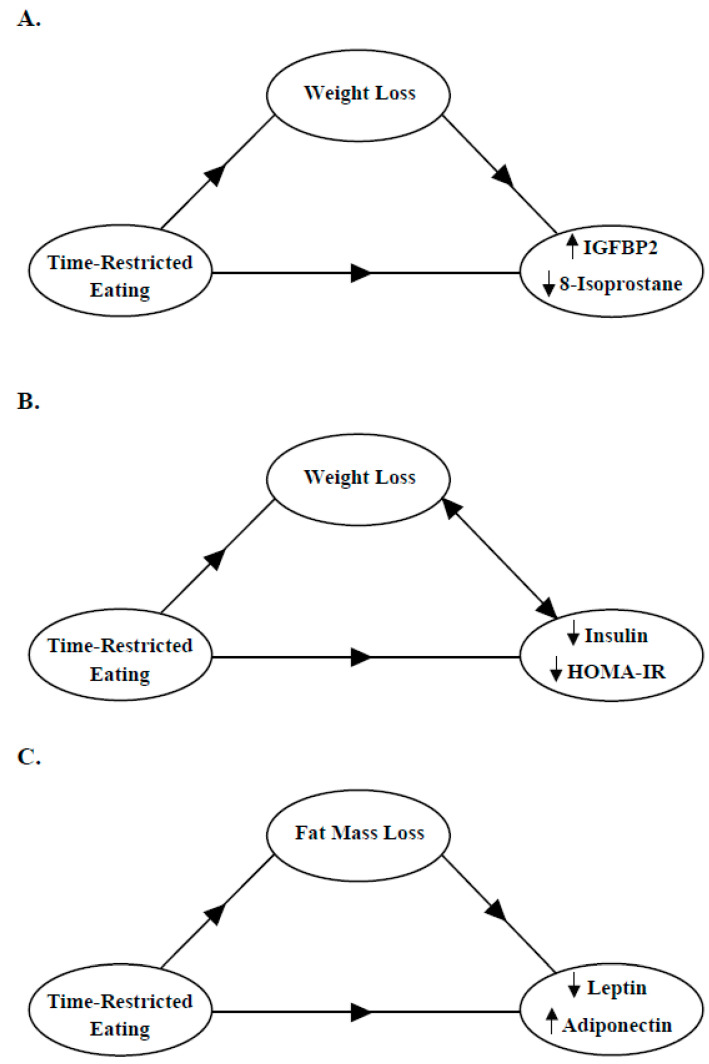
Weight loss as a mediator of the effects of TRE on IGFBP2, 8-isoprostane, insulin, and HOMA-IR. Mediation models presenting (**A**) weight loss as a mediator of the increase in serum IGFBP2 and the decrease in serum 8-isoprostane levels induced by TRE; (**B**) weight loss as a mediator of the decrease in serum insulin and HOMA-IR induced by TRE, and the reversed model where the reduction in serum insulin or HOMA-IR mediate the effect of TRE on weight loss; and (**C**) fat mass loss as mediator of the decrease in serum leptin and the increase in high-molecular-weight adiponectin induced by TRE. The indirect effects of all these models were not significant, suggesting no mediation. IGFBP2: insulin-like growth factor 2; HOMA-IR: homeostatic model assessment of insulin resistance.

Mediation analyses were conducted to test whether weight loss percentage and fat mass loss percentage mediated the effects of TRE on IGFBP2, insulin, HOMA-IR, adipokines, and 8-isoprostane. For IGFBP2, a significant total effect of weight loss was observed (*p*-value < 0.05); however, the direct and indirect effects were not statistically significant, suggesting no mediation (Table 4 and Figure 2A). Furthermore, a post-hoc one-tailed power analysis for the sample size in the mediation analysis revealed a suboptimal power level of 0.64.

Additionally, a statistically significant total effect of TRE on 8-isoprostane was noted, while the direct and indirect effects were not significant, indicating no mediation (Table 4 and Figure 2A). In testing whether weight loss percentage mediated the effects of TRE on insulin and HOMA-IR, significant total and direct effects were observed (*p*-values < 0.05), but the indirect effect was not statistically significant, suggesting no mediation. Since insulin and HOMA-IR can influence weight loss, we reversed the model to test whether the changes in insulin or HOMA-IR mediated the effect of TRE on weight loss. However, no significant mediation was found (Table 4 and Figure 2B). Moreover, we tested whether changes in body composition indicators mediated the changes in IGFBP2, insulin, HOMA-IR, and 8-isoprostane. However, no significant mediation was observed. Furthermore, since leptin correlates with fat mass, mediation analysis was conducted to examine whether leptin changes over time were mediated by fat mass changes. The analysis revealed that the percentage of fat mass loss did not appear to be a significant mediator of the effect of TRE on leptin. Change in fat mass was also not a significant mediator of the effects of TRE on adiponectin (Table 4 and Figure 2C).

## 4. Discussion

This study aimed to investigate the impact of daily 4 to 6 h TRE on circulating growth factors, adiposity markers, and inflammation, examining their dependance on weight loss. Over the eight-week TRE intervention, circulating IGF1, IGFBP1, and IGFBP3 levels showed no significant changes. In contrast, there was a significant effect of group (TRE vs. control) over time on IGFBP2 levels. However, the increase in circulating IGFBP2 levels was only significant in the higher- but not the lower-weight-loss group. Conversely, 8-isoprostane, insulin, and HOMA-IR decreased following the TRE intervention.

Unlike in rodents [17,18], observational and randomized clinical trials have both found that prolonged calorie restriction does not result in a reduction in plasma IGF1 levels [14,15,19]. Consistent with this pattern, the present study indicates that an 8-week, 4–6 h TRE intervention did not significantly change plasma IGF1 levels. Longer fasting periods [20] or a combination of TRE with exercise training may be required to lower plasma IGF1 levels. Indeed, one study found that eucaloric 8 h TRE intervention (10 a.m.–6 p.m.) over 2 and 12 months reduced IGF1 levels in resistance-trained males compared with isocaloric non-TRE controls [11,12]. This reduction was accompanied by a significant but mild decrease in fat mass (average of 1.62 kg), circulating leptin, and IL1β, as well as an increase in adiponectin levels after 2 months. Another eucaloric study by the same investigators found that a 4-week TRE intervention reduced IGF1 levels in elite cyclists compared with non-TRE controls, accompanied by small reductions in body weight (−2%), fat mass percentage (−1.1%), and the ratio of neutrophils to lymphocytes [21]. Conversely, short-term studies found that an 8 h TRE intervention (8 a.m.–4 p.m.) over five weeks in anovulatory women with PCOS also significantly increased IGF1 levels, along with reductions in body weight, fat mass, visceral fat area, waist-to-hip ratio, insulin, HOMA-IR, and hsCRP [13]. The discrepancies in study findings may be related to various factors such as the duration of the intervention, health status, sex, activity levels, and dietary composition. For example, macronutrient intakes are known to influence IGF1 levels [22,23], which must be considered in the design of future trials.

Consistent with some calorie restriction studies [15,24], but not all [19], this study found a small but significant increase in plasma IGFBP2 levels induced by TRE. In the sub-analysis, IGFBP2 levels significantly increased in the higher-weight-loss participants who lost more than 3.5% of their baseline body weights but not in the in the lower-weight-loss participants. Indeed, the changes in IGFBP2 levels negatively correlated with changes in body weight, BMI, and fat mass. These findings suggest that the impact of TRE on IGFBP2 levels is contingent on weight loss and alterations in body composition. While it remains unclear what amount of change in IGFBP2 levels would be clinically significant, these findings suggest that an eight-week TRE intervention may exert a beneficial modest increase in IGFBP2 levels. However, these findings warrant confirmation and further investigation, as the TRE-induced increase in IGFBP2 might entail metabolic significance. For example, a study reported that low IGFBP2 was the strongest predictor for prediabetes (OR: 7.5) in women, while in men, the strongest predictor was IGFBP1 (OR: 13.4) [25]. Additionally, two studies reported that IGFBP2 levels increased following bariatric surgery [26,27], with one reporting that the rise in IGFBP2 correlated with insulin sensitivity, and partially mediated the early metabolic improvements induced by the surgery [27].

While long-term calorie restriction and intermittent fasting are known to cause a sustained increase in plasma IGFBP1 concentrations [14,15], this eight-week TRE intervention did not significantly alter plasma IGFBP1, IGFBP3, or IGF1/IGFBP3 ratio. Furthermore, in the sub-analysis comparing lower- vs. higher-weight-loss participants, there were no significant changes in these biomarkers over time, suggesting that neither TRE nor the concurrent modest weight loss induced by TRE affected these biomarkers. It is possible that the relatively short duration of this intervention leading to modest weight loss might not have been sufficient to cause detectable changes in IGFBPs. Importantly, the molar ratio of IGF1/IGFBP3 was calculated in this study as a proxy for free bioavailable IGF1. Notably, IGFBP3 is the most abundant in human blood compared with other IGFBPs. Hence, it limits the bioavailability of most circulating IGF1 [28]. The findings of this analysis contradict studies reporting that weight loss interventions reduce the IGF1/IGFBP3 ratio [14,15,19,24]. However, given its far greater abundance in the circulation, IGFBP3 is less sensitive than other IGFBPs to being modified by fasting and caloric restriction. Therefore, it was not surprising to see a significant change in only one IGFBP, but not others, following this eight-week daily 4 to 6 h TRE intervention. Thus, longer interventions with intensive caloric restriction are likely to be needed to detect an effect of fasting on IGFBP3. In the future, studies may focus on investigating the effects of a longer TRE intervention on these biomarkers.

In line with other TRE and intermittent fasting trials [29,30], this present study showed a significant reduction in plasma fasting insulin and HOMA-IR following eight weeks of daily TRE. With hyperinsulinemia and insulin resistance being associated with higher risk of cardiovascular disease, diabetes, and cancer [31], the current findings highlight that TRE may confer preventive properties against these chronic diseases. Participants in the TRE group almost reached normal insulin sensitivity (HOMA-IR < 2.5) following the intervention. Moreover, the significant effects of TRE on insulin and HOMA-IR disappeared after adjusting for TRE adherence, but not when adjusting for weight loss percentage. When stratifying participants based on their weight loss percentage, the change in fasting insulin over time was not significant even in the higher-weight loss group. This may suggest that in this study, restricting food intake within a short period may have been a stronger factor in reducing insulin levels than weight loss. It is also possible that stratifying participants according to their weight loss percentage weakened the statistical power. Moreover, while weight loss induced by calorie restriction and endurance exercise is known to powerfully reduce insulin levels and improve insulin sensitivity [32,33,34,35], it is conceivable that the degree of weight loss achieved in this study was not sufficient to emerge as the primary factor responsible for reducing insulin and enhancing its actions. Importantly, some of the beneficial effects of fasting seem to be independent of weight loss. For example, TRE improves insulin sensitivity and metabolic parameters in the absence of weight loss [6,36]. This was demonstrated in a eucaloric clinical trial comparing the effect of TRE with an isocaloric non-TRE control [6,36].

The current study observed no significant impact of TRE on plasma leptin and HMW adiponectin levels across the intervention period. These findings align with existing literature, which has reported inconsistent effects of TRE on adipokine regulation, particularly in short-term interventions [37]. However, in participants with greater weight loss, leptin levels exhibited a significant reduction, consistent with established evidence linking leptin to changes in fat mass [37]. Regarding inflammatory and oxidative stress markers, no significant alterations in hsCRP were detected, in line with previous studies indicating that TRE may not consistently affect systemic inflammation [37]. Notably, however, TRE led to a significant reduction in 8-isoprostane levels, suggesting a potential attenuation of oxidative stress. 

When exploring mediation models, changes in anthropometric variables, including changes in body weight and fat mass, did not emerge as significant mediators of the effects of TRE on circulating IGFBP2, adipokines, insulin, and HOMA. It is important to note that this study was not specifically designed to test the effects of TRE on these biomarkers within the context of mediation models. However, these findings should be interpreted with caution, given the underpowered nature of the sample size used in the mediation analysis in this study.

The present study has several strengths, including being the first to examine the effect of 4 to 6 h TRE on circulating IGF1 and IGF1-binding proteins in men and women with obesity. The effects of 8-week TRE on markers of oxidative stress, such as plasma 8-isoprostane, and glucose metabolism, such as fasting glucose and insulin, HOMA-IR, and Hb1Ac, were also studied to examine their dependance on weight loss. Moreover, the ratio of IGF1/IGFBP3 was calculated to estimate the proportion of bound IGF1. However, the present study also has its limitations. Measuring IGF1 and its binding proteins was not the primary focus of the original study; the sample size was relatively small, especially for mediation analysis; the eight-week intervention period may have been too short to observe the long-term and full range of effects of TRE on the biomarkers of interest; and the controls were more physically active compared with the TRE participants at baseline and throughout the trial period. However, all TRE vs. control analyses were conducted before and after adjusting for step count, and no differences between the results were noted. All these limitations should be considered in future studies to ensure more robust outcomes.

## 5. Conclusions

An eight-week short-term 4 to 6 h daily TRE regimen, resulting in a modest average weight loss of 3.5%, did not exert significant changes on circulating levels of IGF1, IGFBP1, IGFBP3, IGF1/IGFBP3, adiponectin, or hsCRP. However, TRE significantly increased plasma IGFBP2 and decreased plasma leptin in participants who lost more than 3.5% of their baseline body weight. This elevation in IGFBP2 with TRE, potentially impacting the AKT/mTOR pathway, may have implications for cardiovascular disease, diabetes, cancer, and aging. However, these findings must be confirmed in future studies. Furthermore, this TRE intervention improved markers of insulin resistance, an effect that depended on TRE adherence. Moreover, TRE reduced 8-isoprostane levels, an effect that was more pronounced with greater weight loss and was related to loss of fat mass. Additional larger and longer trials are warranted to solidify the understanding of TRE’s effects on these biomarkers.

## Figures and Tables

**Table 1:** Changes in body weight and body composition in participants with obesity after 8-weeks of time-restricted eating (TRE) compared with control.

Variables	TRE (n = 35)			Control (n = 14)			*p*-Value		
	Baseline	Week 8	Change	Baseline	Week 8	Change	Group	Time	Group × Time
Age (years)	47.2 (1.8)			44.9 (2.5)			--	--	--
Sex (female/male)	32/3			12/2			--	--	--
Race or ethnic group									
White	4			2			--	--	--
Black	24			6			--	--	--
Asian	3			2			--	--	--
Hispanic	3			4			--	--	--
Other	1			0			--	--	--
Body composition	(n = 27–35)			(n = 11–14)					
Body weight (kg)	102 (2.9)	98.4 (2.9)	−3.6 (0.3) ***	92.1 (4.6)	92.2 (5)	0.2 (0.5)	0.164	<0.001	<0.001
Fat mass (kg)	48.1 (2.2)	45.9 (2.1)	−2.2 (0.3) ***	41.5 (3.4)	40.8 (3.3)	−0.6 (0.5)	0.151	<0.001	0.006
Lean mass (kg)	51.7 (1.7)	50.3 (1.7) *	−1.4 (0.2) ***	46.6 (2.7)	46.4 (2.7)	−0.3 (0.4)	0.167	<0.001	0.017
Visceral fat mass (kg)	1.36 (0.10)	1.20 (0.09)	−0.16 (0.04) ***	1.11 (0.17)	1.07 (0.14)	−0.04 (0.07)	0.306	0.019	0.127
Height (cm)	165 (0.01)	--	--	160 (0.02)	--	--	0.076	--	--
BMI (kg/m^2^)	37.4 (0.9)	36.1 (0.9) *	−1.3 (0.2) ***	35.9 (1.4)	35.7 (1.4)	−0.2 (0.3)	0.589	<0.001	<0.001
Waist circumference (cm)	97.8 (1.8)	91.0 (1.73)	−6.8 (0.4) ***	93.7 (2.9)	91.0 (2.7)	−2.7 (0.6) ***	0.688	<0.001	<0.001
Adherence and activity	(n = 32–35)			(n = 13–14)					
Adherence (days/week)	--	6.25 (0.10)	--	--	0.0	--	--	--	--
Physical activity (steps/d)	7520 (507)	7288 (531)	−232 (383)	9477 (795) ^#^	9836 (834) ^#^	359 (600)	0.016	0.859	0.412

Data are presented as means (SEM). *p*-value: repeated measures ANOVA with groups (TRE vs. control) as the between-subject factor and time (baseline and week 8) as the within-subject factor. * *p* < 0.05 and *** *p* < 0.001 for TRE-Week 8 vs. TRE-Baseline; ^#^ *p* < 0.05 for TRE vs. control (time-matched) based on multi-comparison test. Body composition components (fat mass, lean mass, and visceral fat mass) were measured using DXA. Physical activity was evaluated in step counts measured over 7 days during the baseline period and at week 8 using wearable fitness tracking devices. BMI: body mass index. Note: Height *p*-value is based on independent sample *t*-test comparing TRE vs. control groups.

**Table 2:** Changes in circulating growth and metabolic factors in participants with obesity after 8 weeks of time-restricted eating (TRE) intervention compared with control.

Circulating Biomarkers	TRE (n = 35)			Control (n = 14)			*p*-Value		
	Baseline	Week 8	Change	Baseline	Week 8	Change	Group	Time	Group × Time
Growth factors	(n = 19–29)			(n = 9)					
IGF-1	153 (12.5)	158 (12.1)	5.03 (7.07)	194 (18.7)	184 (18.0)	−9.39 (10.54)	0.125	0.737	0.267
IGFBP1 (ng/mL)	2.93 (0.60)	2.79 (0.33)	−0.14 (0.48)	2.90 (0.87)	1.74 (0.48)	−1.16 (0.70)	0.472	0.139	0.243
IGFBP2 (ng/mL)	190 (29.9)	226 (26.5)	36.0 (22.7)	261 (43.5)	200 (38.4)	−61.3 (33.0)	0.620	0.534	0.022
IGFBP3 (ng/mL)	4341 (467)	4313 (456)	−27.7 (356)	4375 (696)	3709 (680)	−665.2 (531)	0.712	0.288	0.328
IGF1/IGFBP3 (molar ratio)	0.147 (0.020)	0.152 (0.018)	0.005 (0.016)	0.207 (0.030)	0.225 (0.027) ^#^	0.018 (0.024)	0.042	0.435	0.660
Glucoregulatory factors	(n = 23)			(n = 8)					
Insulin (μU/mL)	14.65 (1.66)	12.41 (1.46)	−2.24 (0.98) *	9.84 (2.82)	14.59 (2.47)	4.75 (1.66) **	0.656	0.202	0.001
Fasting blood glucose (mg/dL)	93.3 (2.26)	89.5 (2.62)	−3.78 (2.12)	93.6 (3.83)	97.2 (4.44)	3.63 (3.60)	0.356	0.970	0.087
HOMA-IR	3.44 (0.42)	2.79 (0.38)	−0.648 (0.29) *	2.26 (0.72)	3.63 (0.65)	1.363 (0.49) *	0.819	0.221	0.001
HbA1C (%)	5.89 (0.11)	5.66 (0.11)	−0.23 (0.05) ***	5.88 (0.18)	5.66 (0.18)	−0.21 (0.08) **	0.980	<0.001	0.879
Adipokines	(n = 20)			(n = 9)					
Leptin (ng/mL)	61.9 (7.2)	52.3 (5.4)	−9.6 (6.2)	40.5 (10.7)	51.3 (8.0)	10.8 (9.3)	0.264	0.916	0.079
HMW adiponectin (ng/mL)	3836 (547)	3996 (511)	160.6 (164)	3908 (815)	4259 (761)	351.3 (245)	0.859	0.094	0.523
Inflammation/oxidative stress	(n = 17, 25, 25, 24)			(n = 9, 10, 10, 10)					
hsCRP (mg/L)	5.51 (0.79)	6.68 (1.67)	1.17 (1.29)	2.74 (1.09)	5.63 (2.29)	2.88 (1.77)	0.332	0.077	0.442
8-isoprostane (pg/mL)	33.9 (2.86)	21.9 (2.82)	−12.00 (2.74) ***	32.6 (4.52)	35.3 (4.47)	2.70 (4.34) *	0.204	0.079	0.007
Interleukin-6	3.96 (0.92)	5.21 (0.91)	1.25 (0.97)	4.5 (1.45)	5.01 (1.44)	0.51 (1.53)	0.906	0.337	0.684
TNF	11.7 (1.78)	11.0 (1.99)	−0.746 (1.63)	11.9 (2.76)	12.1 (3.08)	0.21 (2.53)	0.848	0.860	0.753

Data are presented as mean ± SEM. *p*-values: repeated measures ANOVA with groups (TRE vs. Control) as the between-subject factor and time (baseline and week 8) as the within-subject factor. * *p* < 0.05, ** *p* < 0.01, and *** *p* < 0.001 for week 8 vs. baseline (group-matched); ^#^ *p* < 0.05 for TRE vs. control (time-matched) based on multi-comparison test. To convert IGF1 concentration from ng/mL to nmol/L, multiply by 0.13; to convert IGFBP1 concentration from ng/mL to nmol/L, multiply by 0.22; to convert IGFBP2 concentration from ng/mL to nmol/L, multiply by 0.036; to convert IGFBP3 concentration from ng/mL to nmol/L, multiply by 0.036; to convert insulin concentration from μIU/mL to pmol/L, multiple by 6.945; to convert blood glucose from mg/dL to mmol/L, multiply by 0.0555. IGF-1: insulin-like growth factor 1; IGFBP: IGF-binding protein; HOMA-IR: homeostatic model assessment for insulin resistance; HbA1C: glycated hemoglobin A1C; HMW adiponectin: High-molecular-weight adiponectin; hsCRP: high-sensitivity C-reactive protein.

**Table 3:** Sub-analysis comparing changes in circulating growth and metabolic factors in participants with obesity stratified by weight loss percentage following 8 weeks of time-restricted eating.

Circulating Biomarkers	Lower Weight Loss, ≤3.5% (n = 29)			Higher Weight Loss, >3.5% (n = 20)			*p*-Value		
	Baseline	Week 8	Change	Baseline	Week 8	Change	Group	Time	Group × Time
Body weight (kg)	97.5 (3.3)	96.6 (3.2)	−1.0 (0.3) **	101.5 (4.0)	96.7 (3.8)	−4.8 (0.4) ***	0.670	<0.001	<0.001
Fat mass (Kg)	44.8 (2.4)	43.7 (2.4)	−1.0 (0.3) **	48.3 (3.0)	45.5. (2.9)	−2.8 (0.4) ***	0.495	<0.001	<0.001
Growth factors	(n = 19)			(n = 10)					
IGF-1 (ng/mL)	168 (13.6)	169 (12.7)	0.76 (7.42)	162 (18.8)	162 (17.5)	0.21 (10.23)	0.776	0.939	0.966
IGFBP1 (ng/mL)	3.88 (0.91)	3.02 (0.43)	−0.86 (0.65)	2.71 (1.25)	2.01 (0.59)	−0.70 (0.90)	0.323	0.166	0.887
IGFBP2 (ng/mL)	250 (29.4)	220 (27.3)	−29.9 (23.2)	146 (39.4) ^#^	213 (36.7)	67.1 (31.1) *	0.209	0.346	0.019
IGFBP3 (ng/mL)	4241 (478)	4134 (472)	−106.3 (370)	4562 (659)	4110 (651)	−452.1 (511)	0.844	0.384	0.588
IGF1/IGFBP3 (molar ratio)	0.172 (0.021)	0.176 (0.02)	0.004 (0.017)	0.154 (0.029)	0.173 (0.028)	0.019 (0.023)	0.751	0.426	0.594
Glucoregulatory factors	(n = 20)			(n = 11)					
Insulin (μU/mL)	14.0 (1.84)	14.6 (1.5)	0.585 (1.22)	12.4 (2.48)	10.1 (2.02)	−2.3 (1.65)	0.253	0.410	0.170
Fasting blood glucose (mg/dL)	93.2 (2.42)	93.8 (2.82)	0.600 (2.27)	93.6 (3.27)	87.3 (3.81)	−6.364 (3.06) *	0.451	0.141	0.078
HOMA-IR	3.31 (0.47)	3.44 (0.40)	0.135 (0.362)	2.83 (0.63)	2.22 (0.53)	−0.609 (0.489)	0.208	0.442	0.231
HbA1C (%)	5.93 (0.11)	5.72 (0.11)	−0.21 (0.05) ***	5.80 (0.15)	5.55 (0.15)	−0.25 (0.07) ***	0.431	<0.001	0.664
Adipokines	(n = 19)			(n = 10)					
Leptin (ng/mL)	47.0 (7.2)	51.5 (5.5)	4.5 (6.3)	71.0 (9.9)	52.9 (7.6)	−18.1 (8.7) *	0.194	0.216	0.044
HMW adiponectin (ng/mL)	4091 (556)	4391 (515)	299.8 (168)	3415 (766)	3483 (709)	67.8 (231)	0.387	0.209	0.424
Inflammation/oxidative stress	(n = 17–21)			(n = 10–14)					
hsCRP (mg/L)	4.28 (2.76)	6.59 (4.45)	2.31 (1.98)	10.78 (3.60)	14.80 (5.81)	4.02 (2.58)	0.221	0.062	0.601
8-isoprostane (pg/mL)	33.2 (3.12)	29.5 (3.2)	−3.71 (3.15)	34.0 (3.82)	20.1 (3.92)	−13.93 (3.86) **	0.323	0.001	0.048
Interleukin-6	3.46 (0.99)	5.33 (0.99)	1.876 (1.03)	5.09 (1.21)	4.88 (1.21)	−0.214 (1.26)	0.661	0.316	0.209
TNF	10.8 (1.88)	10.3 (2.11)	−0.49 (1.75)	13.3 (2.4)	12.9 (2.68)	0.423 (2.22)	0.395	0.749	0.981

Data are presented as mean ± SEM. *p*-values: repeated measures ANOVA with groups (≤3.5% vs. <3.5%) as the between-subject factor and time (baseline and week 8) as the within-subject factor. * *p* < 0.05, ** *p* < 0.01, and *** *p* < 0.001 for week 8 vs. baseline (group-matched). ^#^ *p* < 0.05 for lower vs. higher weight loss (time-matched) based on multi-comparison test. To convert IGF1 concentration from ng/mL to nmol/L, multiply by 0.13; to convert IGFBP1 concentration from ng/mL to nmol/L, multiply by 0.22; to convert IGFBP2 concentration from ng/mL to nmol/L, multiply by 0.036; to convert IGFBP3 concentration from ng/mL to nmol/L, multiply by 0.036; to convert insulin concentration from μIU/mL to pmol/L, multiple by 6.945; to convert blood glucose from mg/dL to mmol/L, multiply by 0.0555. IGF-1: insulin-like growth factor 1; IGFBP: IGF-binding protein; HOMA-IR: homeostatic model assessment for insulin resistance; HbA1C: glycated hemoglobin A1C; HMW adiponectin: high-molecular-weight adiponectin; hsCRP: high-sensitivity C-reactive protein.

**Table 4:** Mediators of the effects of 8-week time-restricted eating on circulating biomarkers in participants with obesity.

Circulating Biomarkers	Mediation Models	Indirect Effect				Direct Effect				Total Effect				% Mediation
		Estimate (SE)	β	Z	*p*-Value	Estimate (SE)	β	Z	*p*-Value	Estimate (SE)	β	Z	*p*-Value	
IGFBP2	TRE → Weight Loss % → ∆IGFBP2	−10.3 (46.76)	−0.046	−0.220	0.826	−87.02 (60.57)	−0.385	−1.437	0.151	−97.32 (39.27)	−0.431	−2.478	0.013	10.6%
Insulin	TRE → Weight Loss % → ∆Insulin	1.06 (1.84)	0.087	0.589	0.556	5.91 (2.60)	0.473	2.270	0.023	6.99 (1.89)	0.560	3.701	<0.001	15.2%
	TRE → ∆Insulin → Weight Loss %	0.25 (0.43)	0.051	0.585	0.558	3.22 (0.76)	0.653	4.267	<0.001	3.60 (0.58)	0.671	6.270	<0.001	6.9%
HOMA-IR	TRE → Weight Loss % → ∆HOMA-IR	0.24 (0.55)	0.067	0.446	0.655	1.77 (0.78)	0.481	2.279	0.023	2.01 (0.56)	0.547	2.279	<0.001	11.9%
	TRE → ∆HOMA-IR → Weight Loss %	0.18 (0.41)	0.037	0.444	0.657	3.29 (0.75)	0.667	4.389	<0.001	3.60 (0.57)	0.671	6.270	<0.001	5.0%
8-isoprostane	TRE → Weight Loss % → ∆8-isoprostane	3.21 (3.59)	0.097	0.894	0.371	11.49 (6.05)	0.349	1.900	0.057	14.70 (5.06)	0.446	2.906	0.004	21.8%
Leptin	TRE → ∆FM% → ∆Leptin	742 (4453)	0.018	0.167	0.868	10,432 (9243)	0.253	1.129	0.259	20,471 (11,004)	0.332	1.860	0.063	3.6%
Adiponectin	TRE → ∆FM% → ∆Adiponectin	99.45 (158.1)	0.071	0.629	0.529	74.52 (319.7)	0.053	0.233	0.816	190.7 (289.6)	0.124	0.659	0.510	52.1%

Data are presented as non-standardized effect estimate ± standard error (SE). Betas are completely standardized effect sizes. TRE: time-restricted eating; ∆ symbolizes the change in a variable over time; IGFBP2: insulin-like growth factor-binding protein 2; HOMA-IR: homeostatic model assessment for insulin resistance; ∆FM%: change in fat mass as a percentage compared with baseline.

## Data Availability

Data described in the manuscript may be made available upon request pending application and approval of the corresponding author.

## Supplementary Materials

The following supporting information can be downloaded at: https://www.mdpi.com/article/10.3390/nu16203476/s1, Table S1: Correlation matrix of body composition indicators and circulating biomarkers.
