# Supplementary material for: Effect of Time-Restricted Eating on Circulating Levels of IGF1 and Its Binding Proteins in Obesity: An Exploratory Analysis of a Randomized Controlled Trial
Source: Nutrients. 2024 Oct 14;16(20):3476. doi: 10.3390/nu16203476 (PMC11510611; doi:10.3390/nu16203476)
Supplement: Supplementary file 1 [file nutrients-16-03476-s001.zip › nutrients-3186798-supplementary.pdf]

Table S1. Correlation matrix including body composition indicators and circulating biomarkers.

	ΔIGF-1	ΔIGFBP1	ΔIGFBP2	ΔIGFBP3	ΔIGF1/ IGFBP3	ΔInsulin	ΔHOMA	ΔGlucose	ΔLeptin	ΔAPN	Δ8-isoprostane	ΔCRP	ΔIL6	ΔTNF	Weight Loss %	ΔBMI	ΔWC	ΔFM	ΔVFM	ΔLM
**ΔIGF-1**	—																			
**ΔIGFBP1**	0.051	—																		
**ΔIGFBP2**	0.209	0.006	—																	
**ΔIGFBP3**	0.144	0.458*	-0.189	—																
**ΔIGF1/IGFBP3**	0.271	-0.467*	0.334	-0.824***	—															
**ΔInsulin**	-0.098	-0.13	-0.145	0.08	-0.065	—														
**ΔHOMA-IR**	-0.142	-0.191	-0.128	0.074	-0.053	0.978***	—													
**ΔGlucose**	-0.128	-0.252	-0.04	0.009	0.031	0.619***	0.712***	—												
**ΔLeptin**	0.307	-0.082	-0.071	-0.072	0.22	0.183	0.137	-0.048	—											
**ΔAPN-HMW**	0.194	-0.124	-0.237	-0.197	0.244	0.106	0.118	0.113	0.105	—										
**Δ8-isoprostane**	-0.151	0.027	-0.324	0.138	-0.155	0.28	0.3	0.321	0.227	0.006	—									
**ΔhsCRP**	0.089	0.167	0.086	-0.093	0.049	-0.202	-0.224	-0.1	-0.275	0.066	0.047	—								
**ΔIL6**	-0.192	0.033	0.005	0.116	-0.124	-0.045	-0.04	0.138	-0.401*	-0.136	0.065	0.149	—							
**ΔTNF**	0.019	0.175	-0.094	-0.018	0.041	-0.019	-0.041	-0.179	0.086	0.003	0.153	0.071	-0.157	—						
**Weight Loss %**	-0.023	-0.254	-0.356	-0.112	0.145	0.456**	0.433*	0.335	0.391*	0.184	0.37*	0.104	0.044	0.063	—					
**ΔBMI**	-0.144	-0.027	-0.392*	-0.046	-0.039	0.286	0.268	0.187	0.276	0.367	0.303	0.277	0.108	0.182	0.712***	—				
**ΔWC**	-0.128	-0.394*	-0.328	-0.191	0.168	0.313	0.324	0.367*	0.337	0.156	0.444**	0.208	0.115	-0.064	0.527***	0.319*	—			
**ΔFM**	-0.48*	-0.072	-0.427*	-0.006	-0.215	0.205	0.227	0.135	0.075	0.229	0.391*	0.219	0.205	-0.038	0.674***	0.78***	0.406*	—		
**ΔVFM**	-0.393	-0.152	0.021	-0.092	-0.136	0.073	0.074	-0.224	0.207	0.228	0.221	0.063	-0.095	-0.173	0.233	0.33*	0.242	0.554***	—	
**ΔLM**	0.2	0.137	0.023	0.211	-0.093	0.296	0.231	0.322	0.373	-0.277	0.197	0.134	0.13	-0.281	0.44**	0.342*	0.11	0.195	-0.022	—

Data are presented as Pearson's correlation coefficient. *P<0.05, **P<0.01, ***P<0.001.
Δ symbolizes the change in a variable over time. IGF1: Insulin-like growth factor; IGFBP1: IGF-binding protein-1; IGFBP2: IGF-binding protein-2; IGFBP3: IGF-binding protein-3; HOMA-IR: Homeostatic model assessment for insulin resistance; APN-HMW: Adiponectin (High-Molecular Weight); hsCRP: High sensitivity C-reactive protein; IL6: Interleukin-6; TNF: Tumor necrosis factor; BMI: Body mass index; WC: Waist circumference; FM: Fat mass; VFM: Visceral fat mass; LM: Lean mass.
